# A narrative review of the moderating effects and repercussion of exercise intervention on osteoporosis: ingenious involvement of gut microbiota and its metabolites

**DOI:** 10.1186/s12967-022-03700-4

**Published:** 2022-10-27

**Authors:** Yuan-Wei Zhang, Mu-Min Cao, Ying-Juan Li, Xiang-Xu Chen, Qian Yu, Yun-Feng Rui

**Affiliations:** 1 Department of Orthopaedics, School of Medicine, Zhongda Hospital, Southeast University, No. 87 Ding Jia Qiao, 210009 Nanjing, Jiangsu PR China; 2 Multidisciplinary Team (MDT) for Geriatric Hip Fracture Management, School of Medicine, Zhongda Hospital, Southeast University, Nanjing Jiangsu, PR China; 3 School of Medicine, Southeast University, Nanjing, Jiangsu PR China; 4 Orthopaedic Trauma Institute (OTI), Southeast University, Nanjing, Jiangsu PR China; 5 Department of Geriatrics, School of Medicine, Zhongda Hospital, Southeast University, Nanjing, Jiangsu PR China; 6 Department of Gastroenterology, School of Medicine, Zhongda Hospital, Southeast University, Nanjing, Jiangsu PR China

**Keywords:** Exercise intervention, Bone, Osteoporosis, Gut microbiota, Metabolites

## Abstract

Osteoporosis (OP) is a systemic bone disease characterized by the decreased bone mass and destruction of bone microstructure, which tends to result in the enhanced bone fragility and related fractures, as well as high disability rate and mortality. Exercise is one of the most common, reliable and cost-effective interventions for the prevention and treatment of OP currently, and numerous studies have revealed the close association between gut microbiota (GM) and bone metabolism recently. Moreover, exercise can alter the structure, composition and abundance of GM, and further influence the body health via GM and its metabolites, and the changes of GM also depend on the choice of exercise modes. Herein, combined with relevant studies and based on the inseparable relationship between exercise intervention-GM-OP, this review is aimed to discuss the moderating effects and potential mechanisms of exercise intervention on GM and bone metabolism, as well as the interaction between them.

**Supplementary Information:**

The online version contains supplementary material available at 10.1186/s12967-022-03700-4.

## Introduction

Osteoporosis (OP) is a kind of systemic metabolic bone disease characterized by descending bone mass and destruction of bone microarchitecture, predisposing to the enhanced bone fragility and associated fractures, as well as the high disability rate and mortality [1–3]. According to previous reports, 1/3 of female and 1/5 of male over the age of 50 are likely to experience the osteoporotic fractures at least once in their whole lifetime. Meanwhile, the number of the osteoporotic fractures worldwide exceeds 8.9 million every year, and an average of one case of osteoporotic fracture occurs every 3 s, which becomes a major challenge for the patients themselves, their families and social medical security system [4, 5].

Currently, a variety of drugs with different mechanisms of action have been applied in preventing and treating OP. Nevertheless, in the process of integrated management of OP, the pivotal contribution of exercise intervention still cannot be ignored [6, 7]. In the context of current global aging, the middle-aged and elderly individuals, as a special group, are mostly combined with one or more chronic diseases (including OP, diabetes, cardiovascular diseases, osteoarthritis, and so on) [8, 9]. Cardiovascular diseases and OP together account for most of the morbidity and mortality in aging population despite significant improvements in treatment. Recently, converging lines of evidence suggest that these 2 diseases share an etiologic factor, that hyperlipidemia contributes not only to atherosclerotic plaque formation, but also to bone loss, following a similar biologic mechanism involving the lipid oxidation [10]. Meanwhile, dyslipidemia is also a major cause of non-alcoholic fatty liver disease (NAFLD). NAFLD and OP are strictly linked, as more recently it has been evidenced that NAFLD is related to an increased incidence of OP and bone fractures [11]. Although the mechanisms remain poorly understood, it is reasonable to conclude that low levels of physical activity may lead to a decrease in bone mineral density (BMD), development of insulin resistance, and as a consequence to the presence of NAFLD [12]. However, moderate exercise has vital benefits for the middle-aged and elderly individuals to manage several kinds of chronic diseases [13, 14]. Moreover, exercise is also one of the most common, reliable and cost-effective interventions for the prevention and treatment of OP [15]. Especially in the case that the activities of elderly individuals are reduced due to various reasons, how to conduct the exercise intervention that adapted to their own physical health and surrounding environment, and how to ensure the sufficient exercise amount are of great significance.

In addition, as the largest and most complicated micro-ecosystem in human body, the gut microbiota (GM) and its metabolites play a critical role in the regulation of host metabolism, nutrition, immunity, and so on [16]. Meanwhile, more and more researches have showed that the abnormal physiological metabolism of body is not only modulated by its own genes, but also inseparable from the maintenance of GM homeostasis [17]. Changes in the internal and external environment of the body could disrupt the balance between GM and body, resulting in a variety of inflammatory or metabolic diseases, such as inflammatory bowel disease, colon cancer, obesity, diabetes, OP and so on [18–21]. With regard to this, in our previous critical reviews, based on the concept of “brain-gut-bone” axis, we have summarized the modulatory effects and implication of GM to OP [22], as well as the regulative effects and repercussion of the probiotics and prebiotics on OP [2]. In view of the close association between GM and bone metabolism, it is acknowledged that GM might be regarded as a potential target for the prevention and treatment of OP.

Importantly, exercise is of great significance for the maintenance of whole-body health. On one hand, exercise can directly act on various tissues and organs of the body to regulate different functions. On the other hand, exercise could significantly alter the structure, composition and abundance of GM, and further affect the body health through GM and its metabolites [23]. Meanwhile, exercise is able to alter the composition and function of GM, and the changes of GM also depend on the choice of exercise modes [24]. Herein, combined with relevant researches and based on inseparable relationship between exercise intervention-GM-OP, this review is aimed to discuss the moderating effects and potential mechanisms of exercise intervention on GM and bone metabolism, as well as the interaction between them.

## The inseparable association between OP and GM

As the largest micro-ecosystem in human body, more than 10^14^ orders of magnitude bacteria are colonized in the intestine, and the number of genes in the genome of GM is about 150 times that of the total number of genes in human genome [25]. The GM is interdependent with human body, influencing multiple systems (including enteric nervous system, enteroendocrine system, immune system and so on), and playing an indelible role in food digestion, nutrients intake, resistance to the invasion of foreign pathogenic microorganisms, and so on [26]. Currently, the correlation between intestinal microecology and bone metabolism has gradually become a research hotspot all over the world [27]. Especially in the recent years, it has been observed that the changes of GM are closely related to the reduction of bone mass and the incidence of OP in middle-aged and elderly individuals [5, 28]. Meanwhile, more and more human and animal studies reveal that GM may alter the relative activities of osteoblasts and osteoclasts by modulating metabolites, host metabolism, inflammatory reaction, immune response, and other mechanisms, so as to participate in the regulation of bone metabolism.

Specifically, in terms of the clinical studies, Wang et al. [29] conducted the 16 S ribosomal RNA (16 S rRNA) sequencing analysis of the feces from patients with OP, patients with osteopenia and the individuals in the control group, and observed that the composition and diversity of GM in OP group and control group were significantly different, mainly reflected in the proportion of *Firmicutes* and *Bacteroidetes*. Fuhrman et al. [30] examined 60 randomly selected healthy postmenopausal women and revealed that the fecal microbial diversity and relative abundance of *Clostridium* and *Bacillus* were positively related to urinary estrogen metabolites, and the diversity of GM also increased with the enhancement of proportion of hydroxylated estrogen metabolites in urine. Meanwhile, the use of probiotics and prebiotics could change the composition, structure and abundance of GM, indicating the benefits of probiotics and prebiotics for bone metabolism. Regarding this, a previous randomized controlled trial conducted by Nilsson et al. [31] suggested that the postmenopausal women aged 75–80 years old who received a daily dose of 1⊆10^10^ CFU of *Lactobacillus reuteri ATCCPTA 6475* had a significantly lower reduction in BMD than individuals in control group after 12 months of treatment. Lei et al. [32] observed in a prospective randomized controlled trial that the elderly patients with distal radius fractures can also accelerate the process of fracture healing by ingesting *Lactobacillus casei Shirota* for a period of 6 months. Moreover, van den Heuvel et al. [33] showed in a randomized crossover study that the supplementation of *Transgalactooligosaccharides* was able to enhance the intestinal calcium absorption in postmenopausal women, thus enhancing the bone mass and preventing OP.

As for the animals’ studies, by constructing the mice model of postmenopausal OP induced by ovariectomy (OVX), Tu et al. [34] revealed that the estrogen-deficiency was related to the imbalance of GM, impaired intestinal mucosal barrier function, and enhanced inflammatory and immune reactivity. On the basis of this, the metabolites of intestinal pathogenic bacteria could enter into the body circulation through impaired intestinal mucosal barrier, induce the immune reaction mediated by CD4 + T cells, generate a variety of pro-osteoclastogenic cytokines, and mediate the activation of osteoclasts and accelerate the bone resorption. Additionally, Sjögren et al. [35] observed in an animal experiment study that compared with the normal mice in control group, the germ-free (GF) mice had enhanced bone mass and lower levels of CD4 + T cells and tumor necrosis factor-α (TNF-α) in bone marrow. However, after the transplantation of GM from normal mice to GF mice, the bone mass, levels of CD4 + T cells and TNF-α of GF mice returned to normal. Goto et al. [36] indicated that transplanting segmental filamentous bacteria (SFB) into GF mice could increase the number of T helper cell 17 (Th17) cells, resulting in the enhancement of the levels of TNF-α, interleukin 1β (IL-1β), and IL-17, and inducing the expression of receptor activator of nuclear factor-κ B ligand (RANKL), thereby playing a vital role in promoting the formation of osteoclasts. Interestingly, IL-17 also plays a key role in atherosclerosis following obesity-related NAFLD [37]. Yan et al. [38] also reported that the colonization of GM in mice increased the rate of bone formation and length of femur, and detected the enhancement of serum insulin-like growth factor-1 (IGF-1) and bone formation marker (procollagen type I N-terminal propeptide (P1NP)), suggesting that the stimulation of bone formation through GM may be mediated by IGF-1. Zhang et al. [39] showed that fecal microbiota transplantation (FMT) plays an active role in remodeling the GM and improving the bone loss in OVX-induced mice with OP. In details, by correcting imbalance of GM, improving the level of SCFAs, optimizing the intestinal permeability and inhibiting the release of pro-inflammatory cytokines, FMT may inhibit the excessive generation of osteoclasts and obtain the balance between bone formation and bone absorption, thus ameliorating the bone loss in OVX-induced mice with OP.

Taken together, there is a close link between GM and OP, and a healthy intestinal microenvironment state has a significant protective effect on the bone. Importantly, GM mainly affects the balance between osteoblast-mediated bone formation and osteoclast-mediated bone resorption, which plays a vital role in the regulation of bone metabolism and provides a novel target of intervention for the prevention and treatment of OP. Nevertheless, the relevant studies on the association between GM and OP are still in the initial stage, and more researches are needed to further clarify its deep-level mechanisms and explore more effective conditioning measures.

## The moderating effects of exercise intervention on the human and experimental animals’ GM

**Fig. 1:**
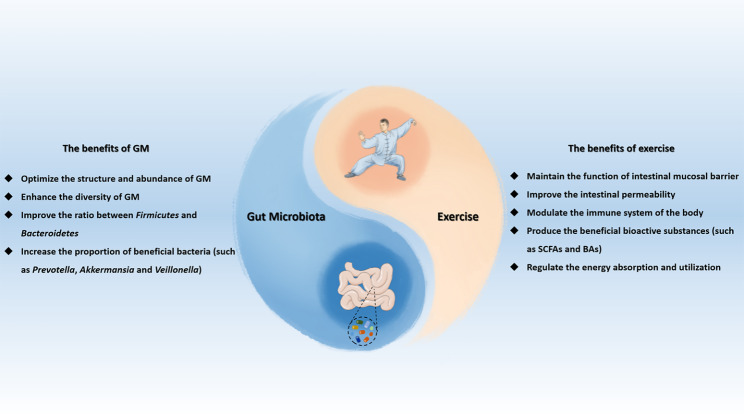
The close interaction relationship between GM and exercise intervention. GM, gut microbiota; SCFAs, short chain fatty acids; BAs, bile acids

As a kind of homeostatic stimulus, exercise intervention may diversify the GM and increase the number of beneficial microbial communities in gut. Exercise intervention contributes to enhancing the gastrointestinal peristalsis of body and promotes the timely excretion of feces, so as to reduce the contact time of intestinal mucosa with pathogens and harmful substances, further affect the characteristics of the intestinal contents and alter the composition of GM [40, 41]. Meanwhile, when participating in different types of exercise, different qualitative and quantitative changes may occur in GM, thereby influencing multiple body functions, such as the nutrient absorption, energy distribution, immune and inflammatory regulation [42]. Moreover, a variety of previous studies have also showed that the exercise intervention might modulate the occurrence and progression of OP and other skeletal diseases by altering intestinal mucosal barrier function and the composition, structure and abundance of GM [43–46]. Accordingly, different kinds of physical activities may also have different influence on the GM [47]. The relationship between exercise intervention and GM is complicated, which depends on several factors, such as the types of exercise, intensities of exercise, duration of exercise, frequency of exercise, surrounding environment during exercise, and so on [48, 49]. Understanding the various functions of GM in exercise performance is of great significance to both ordinary individuals and athletes seeking to improve the exercise effects and reduce the training recovery time. Herein, the moderating effects of exercise intervention on the human and experimental animals’ GM are summarized as follow, and Fig. 1 exhibits the close interaction relationship between the GM and exercise intervention.

### As for human

#### Long-term chronic moderate-to-low intensity exercise

The dynamic balance between GM and host physiological function determines the possibility of host morbidity caused by GM imbalance. However, several previous studies have revealed that long-term chronic moderate-to-low intensity exercise has beneficial effects on body [50, 51]. Moreover, the World Health Organization (WHO) has previously established the guidelines of 150 min of moderate intensity exercise per week as the recommended minimum level of exercise, which seems to be sufficient to alter the composition and abundance of GM and promote the health [52]. Balducci et al. [53] showed that long-term sustained aerobic exercise and resistance exercise with moderate-to-low intensity could reduce the expression of inflammatory cytokines (such as IL-1β and TNF-α), while increase the expression of anti-inflammatory cytokines (such as IL-4 and IL-10) in the body. Meanwhile, according to previous reports, especially in partial professional athletes, the acute and vigorous exercise might induce the transient gastrointestinal symptoms (such as nausea, diarrhea, gastrointestinal bleeding, and so on) [54–56]. However, the participation in normal moderate intensity exercise is considered to resist intestinal inflammatory diseases, and the gastrointestinal disorders usually do not occur after the moderate intensity exercise, which might be associated with the involvement of GM in regulation [57]. Furthermore, the stimulation of sustained moderate intensity exercise also has an indelible effect on maintaining the abundance of GM that can generate the short chain fatty acids (SCFAs) and regulating the expression levels of related genes [58]. Allen et al. [59] observed in a population-based study that after 6 weeks of aerobic endurance training, the contents of propionic acid and butyric acid in the feces, the abundance of butyric acid-producing bacteria, and the relative contents of butyric acid-producing genes, returned to the baseline level after 6 weeks of returning to sedentary life.

In addition, compared with the sedentary women, the premenopausal women who exercise continuously at the low intensity are equipped with the increased abundance of *Akhmann mucophilus* and *Clostridium praevia*, and these bacteria have been verified to be anti-inflammatory bacterial species [60]. Moreover, previous population-based research conducted by Munukka et al. [61] also indicated that the relative abundance of *Verrucomicrobia*, *Verrucomicrobiaceae*, *Akkermansia* and *Dorea* in the feces of the sedentary and overweight women increased after 6 weeks of moderate-to-low intensity power cycling training. Therein, *Akkermansia* and *Dorea* were verified to be able to ferment with dietary fiber as the substrate to generate the butyric acid. Besides, Hamasaki et al. [62] also concluded that as a moderate intensity exercise involving deep breathing and meditation, Tai Chi might induce the beneficial changes in GM and its metabolites by modulating the hypothalamic-pituitary-adrenal (HPA) axis. Similarly, the Chinese traditional martial arts are also a common moderate intensity exercise that is suitable for the physical exercise of middle-aged and elderly individuals. A number of previous studies have revealed that martial arts can effectively optimize the structure and abundance of GM in the middle-aged and elderly individuals [63, 64]. For the middle-aged and elderly individuals who have been practicing the martial arts for a long time, the abundance of *Enteric acidophilus*, *Bifidobacteria* and *Lactobacillus* in their intestines increases, resulting in a decrease of the content of malondialdehyde and an increase of the activity of antioxidant enzyme, so as to improve a variety of metabolic processes in the body and then promote health [65, 66]. Hence, it is acknowledged that appropriate (mainly moderate-to-low intensity) exercise is able to produce the benign changes to the health of human body by regulating the GM and its metabolites.

#### Acute/vigorous exercise

Nevertheless, different from the moderate-to-low intensity exercise, in the case of acute/vigorous exercise, the ischemic effects of intestinal mucosa and the enhancement of intestinal permeability resulting in the intake of bacteria and toxins could induce the inflammatory response of body [67]. According to previous reports, 20–50% of athletes might experience multiple gastrointestinal symptoms that increase with the exercise intensity [68, 69]. In a triathlon competition, Jeukendrup et al. [70] observed 29 well-trained male athletes, and the results suggested that 93% of the participants reported gastrointestinal dysfunction, and two participants abandoned the competition due to the severe vomiting and diarrhea. Hence, it is recognized that gastrointestinal symptoms are more common in athletes, because the body temperature rises during vigorous exercise and blood flows from the gastrointestinal tract to the surrounding muscles and organs (such as heart, liver and lungs) [71, 72]. The redistribution of blood flows away from the intestine and the thermal injury to intestinal mucosa can result in the destruction of intestinal mucosal barrier and then trigger the inflammatory response [73].

With regard to this, Zouhal et al. [74] suggested that the individuals who exercise at the condition of 70% VO_2max_ might reduce the visceral blood flow by 60–70%, and when the blood flow was reduced by 50%, the exercise-induced ischemia may result in the increased intestinal permeability. On the basis of this, the blood flows from the gastrointestinal tract via surrounding organs may also result in the loosening of tight junction proteins (mainly expressed by zonula occludens-1 (ZO-1) and Occludin), so as to further damage the intestinal mucosal barrier [75]. Moreover, in a study of healthy young adult male cyclists who engaged in 4–10 h of endurance exercise per week, Duijghuijsen et al., [76] observed that if they conducted the exercise for one hour with only 70% of maximum workload, they would have insufficient visceral perfusion, which resulted in the decline of gastrointestinal circulation, the increase of intestinal permeability and the damage of accessory organs. Therefore, it is recognized that the acute/vigorous exercise may cause the partial negative effects on human body (mainly gastrointestinal symptoms), which especially needs to be paid enough attention in the future researches.

### As for experimental animals

#### Autonomic exercise

Autonomous exercise is a significant part of the structure of exercise modes. With regard to this, Matsumoto et al. [77] first observed in a study that the composition and abundance of GM were significantly different between the rats in autonomic exercise group and sedentary group. Moreover, other researchers tested the concentration of butyric acid in the cecum of rats, and results revealed that compared with the rats in sedentary group, the concentration of butyric acid in autonomic exercise group was twice that of the former [78, 79]. As one kind of significant SCFAs, the butyric acid provides the energy for colonic epithelial cells and has been verified to have multiple benefits to host. Moreover, wheeled running is one of the most convenient approaches to simulate the autonomous exercise in animal models, and the exercise amount can be calculated by the rotation distance of wheel. Aoki et al. [80] revealed that daily wheeled running can affect the composition of GM in mice, and ameliorate high-fat diet (HFD)-induced obesity by altering the ratio between *Firmicutes* and *Bacteroidetes*. Li et al. [81] also suggested that compared with the HFD-induced osteoarthritis mice in sedentary group, level of lipopolysaccharide (LPS) in circulation of mice (autonomous exercise group) decreased, and the diversity of GM increased, which alleviated the process of chronic inflammation and osteoarthritis in body. These studies have revealed that the composition and diversity of GM could be optimized to varying degrees after the continuous autonomic exercise in the animal models, and the conditions of obesity and metabolic skeletal diseases could be effectively alleviated. The benign changes of GM induced by autonomic exercise might be another effective approach to alleviate or treat the metabolic diseases, but the interaction between autonomic exercise and GM, control parameters of exercise volume, and participation of dietary factors still need to be further explored in the future.

#### Forced exercise

In animal models, the most common type of forced exercise is the forced treadmill exercise. Compared with the autonomous exercise, the forced exercise has significantly different influence on the GM of experimental animals. With regard to this, in the mice model of colitis, Cook et al. [82] observed that the autonomic exercise could attenuate the intestinal inflammation, while the forced exercise exacerbated research outcomes, indicating that different exercise modes might have different influences on the clinical outcomes and composition and abundance of GM during the process of inflammatory injury. Based on this, Allen et al. [83] collected the feces of mice in the autonomous exercise group and forced treadmill exercise group for analysis, and the results showed that both autonomous exercise and forced treadmill exercise altered various individual bacterial taxa, and the level of *Agrobacterium* (closely related to immune function and intestinal diseases) reduced significantly in autonomous exercise group, suggesting that exercise intensity and exercise amount might be the variables influencing the function of GM. In addition, the changes in GM caused by exercise also seem to depend on the physiological state of individuals. A previous study conducted by Gordon et al. [84] indicated that whether the obese-hypertensive rats or normal rats, regular forced exercise may have different influence on the composition and abundance of respective GM. Collectively, there are certain differences in the effects of different exercise intensities and amounts on the changes of GM, and the changes of GM induced by exercise are different in various phenotypic animal models. Further exploration of appropriate exercise modes and the load in different kinds of phenotypic animal models might provide a relatively complete theoretical basis for the establishment of exercise strategies and formulation of exercise prescriptions to a certain extent.

## Current researches and evidence on exercise intervention-GM-OP

The musculoskeletal system plays an indelible role and significance in the human health. In addition to acting as a scaffold for body, it also continuously communicates with other organs through the biochemical signals, and has basic neural, immune and endocrine regulatory functions [85, 86]. The GM and its metabolites are associated with a variety of diseases in musculoskeletal system, including the sarcopenia, osteoarthritis, ankylosing spondylitis, OP, and rheumatoid arthritis [87–91]. The GM is involved in regulating the balance of musculoskeletal development and homeostasis, and related researches have also shifted from describing simple correlations to seeking intervention mechanisms through the basic researches and clinical trials, which has made this field develop rapidly in recent years [92, 93]. Additionally, the role of exercise in promoting the bone health, improving the bone metabolism and preventing and treating OP has been widely verified, and the studies on the mechanisms of exercise in preventing and treating OP has also been a research hotspot in academic circle [94, 95]. Previous studies have showed that the alterations of exercise-induced hormones, cytokines, bone metabolic signaling pathways and mechanical stress are the main mechanisms of exercise in prevention and treatment of OP, while the interaction between exercise intervention, GM and OP has a pivotal position and still cannot be ignored [96–98].

Recently, there have been research reports that the promoting effects of exercise on the bone is closely associated with GM. In a previous study, McCabe et al. [44] focused on how exercise affects the regulation of HFD on the bone of mice. The researchers intervened the mice with HFD or low-fat diet (LFD), and divided mice into autonomous exercise group and sedentary group for a 14-week experimental period. The results of this study indicated that HFD resulted in the decrease of vertebral and tibial trabeculae, the increase of fat in bone marrow, and the disorder of GM in mice. Nevertheless, exercise can improve the adverse symptoms caused by HFD. Specifically, exercise can reduce the disorder of GM caused by HFD, which was conducive to remodeling the structure and abundance of GM. On the basis of this, the researchers observed that exercise can reduce the ratio of *Firmicutes* and *Bacteroides*. Moreover, in terms of the clinical practice, Ilesanmi-Oyelere et al. [43] conducted a randomized controlled trial of the effects of synbiotic supplements (acting by modulating GM) and exercise on the postmenopausal OP in New Zealand. The researchers expected that the combination modes of synbiotic supplements and exercise may be a promising non-invasive method to manage and/or improve the bone health in postmenopausal women. Shimizu et al. [46] concluded that the moderate exercise and proper dietary intake are of great significance for the prevention and treatment of OP. Moreover, by integrating various interventions that modulate the composition and/or diversity of GM, it should be a promising strategy for maintaining personal health status and activities of daily living. Collectively, these results suggested that the improvement of GM by exercise may be a novel mechanism for the exercise to promote bone health, and provided a new direction for the mechanism-related studies of exercise intervention in prevention and treatment of OP. However, to date, there are still few studies on the interaction between exercise intervention-GM-OP, and its in-depth mechanisms and possible intervention strategies need to be further clarified in future. Furthermore, the literatures exhibiting outcomes and involved mechanisms with the application of exercise intervention to bone-related diseases in population-based studies and animals researches from the perspective of GM and its metabolites are summarized in Table 1 [99–101].

**Table 1:** Literatures exhibiting outcomes and involved mechanisms with the application of exercise intervention to bone-related diseases in population-based studies and animals researches from the perspective of GM and its metabolites

Research subjects	Intervention mode	Exercise duration	Research outcomes	Involved mechanisms	References
Population: Previously sedentary overweight women	Moderate-to-low intensity power cycling training	6 weeks	Six-weeks endurance exercise, without changing diet, affected the GM and systemic metabolites of overweight women	The GM, especially *Akkermansia* and *Proteobacteria*, were the exercise-responsive taxa	Munukka et al. (2018)
Population: Postmenopausal women	Combination of isoflavone and exercise	24 weeks	The combination of isoflavones and exercise exhibited favorable effects on serum lipid and body composition of postmenopausal women	The preventive effects of isoflavones on bone loss depended on the individual’s GM for equol production	Wu et al. (2006)
Population: Postmenopausal women	Combination of synbiotic supplements and exercise	12 weeks	The combination of synbiotic supplements and exercise might serve as a noninvasive approach to manage and/or improve body composition and bone health in postmenopausal women	The combination of synbiotic supplements and exercise may promote the growth of beneficial bacteria, increases the level of SCFAs, reduce the inflammatory cytokines, and improve the effects of anti-inflammatory cytokines	Ilesanmi-Oyelere et al. (2021)
Population: Lean and obese women	Supervised and endurance-based exercise training	6 weeks	Exercise-induced alterations of the GM were dependent on obesity status, and exercise increased fecal concentrations of SCFAs in lean, but not obese participants.	Exercise training induced the compositional and functional changes in the human GM that were dependent on obesity status, independent of diet and contingent on the sustainment of exercise.	Allen et al. (2018)
Animal models: HFD-induced OP mice	Sedentary/Autonomous exercise	14 weeks	Exercise prevented the negative effects of HFD on skeletal health	Exercise altered the HFD-induced changes on the composition of GM by reducing the ratio of *Firmicutes* and *Bacteroides*	McCabe et al. (2019)
Animal models: PTOA rats	Sedentary/Treadmill-walking	8 weeks	Treadmill-walking was effective at maintaining the integrity of cartilage-subchondral bone unit and reducing the elevated systematic inflammation factors and microbiome-derived metabolites	The exercise-induced modification of disease-relevant microbial shifts was potentially involved in the mechanisms of exercise-induced amelioration of PTOA	Hao et al. (2022)
Animal models: HFD-induced OA rats	Combination of prebiotic fiber supplements and exercise	12 weeks	The combination of prebiotic fiber supplements and exercise prevented the knee joint damage	The prevention of knee joint damage was related to the normalization of insulin resistance, leptin levels, dyslipidemia, GM and endotoxemia	Rios et al. (2019)
Animal models: HFD-induced OA mice	Sedentary/Wheel-running exercise	4 weeks	Exercise contributed to the relief of chronic inflammation and OA	Exercise reshaped the GM, reduced the LPS levels in the blood and synovial fluid and TLR4 and MMP-13 expression levels, and ameliorated the cartilage degeneration	Li et al. (2021)

Note: OP, osteoporosis; GM, gut microbiota; HFD, high-fat diet; SCFAs, short chain fatty acids; PTOA, post-traumatic osteoarthritis; OA, osteoarthritis; LPS, lipopolysaccharide; TLR4, toll like receptor 4; MMP-13, matrix metallopeptidase 13

## The potential mechanisms of exercise intervention on bone metabolism from the perspective of GM and its metabolites

### The regulation of intestinal metabolites

As one of the significant metabolites of GM, SCFAs are mainly composed of the carboxylic acids and small hydrocarbon chains [102, 103]. SCFAs is mainly composed of the acetic acid, propionic acid, butyric acid, and so on. Therein, the acetic acid and propionic acid can act on the liver and surrounding organs, and are the substrates for gluconeogenesis and lipogenesis, while the butyric acid provides the energy for colonic epithelial cells [104–106]. Barton et al. [107] compared the 16 S rRNA sequencing and metabolomics results of 40 professional athletes and 46 sedentary controls, and found that the GM of professional athletes had enhanced the ability to synthesize amino acids and regulate the carbohydrate metabolic pathways, and the contents of metabolites also increased significantly. Yu et al. [108] revealed that increased production of metabolites after exercise can induce an increase in the genetic expression of sestrin-2 (SESN2) and CREB regulated transcription coactivator 2 (CRTC2) in the liver of mice, resulting in a decline in the expression of inflammatory factors (such as IL-1β and TNF-α), and significantly reduce the level of LPS in circulation, so as to ameliorate the inflammatory or metabolic related diseases. Mailing et al. [109] also showed a role of butyric acid in the intestine to prevent the degradation of intestinal mucosa. Researchers observed an increase in relative abundance of butyric acid-producing bacteria (*Blautia*, *Roseburia*, *Anaerostipes*, *Butyricicoccus*) in the intestine of athletes under high intensity exercise conditions. Butyric acid can directly supply the energy to the intestinal epithelial cells and repair injury, thereby reducing the injury to intestine of body caused by high intensity exercise training [110, 111]. Meanwhile, Xia et al. [112] suggested that the benefits of exercise could be transmitted between the individuals via the transplantation of GM. Specifically, after 12 weeks of moderate intensity exercise, the systolic blood pressure of hypertensive rats reduced, accompanied by the increase in diversity of GM, and this antihypertensive effect can be transmitted to non-exercise hypertensive rats by means of FMT. Scheiman et al. [113] transplanted the atypical *Veillonella* isolated from the feces of marathon athletes into experimental mice, and the exercise endurance of mice was significantly improved. Besides, several studies have also reported that the production of butyric acid is related to the production of heat shock protein 70 (HSP70) [114, 115]. HSP70 contributes to maintaining the structural and functional properties of intestinal epithelial cells in response to the damage caused by the prolonged vigorous exercise, which may provide structural and functional stability to intestinal epithelial cells under adverse conditions. Hence, these researches revealed that GM and SCFAs played a critical role in mediating the exercise benefits.

In addition to SCFAs, the metabolic disturbance of bile acids (BAs) is also involved in the occurrence and progression of OP. As a kind of signal molecule, BAs not only play a significant role in the absorption, transport and distribution of fat and fat-soluble vitamins, but also participate in the regulation of energy metabolism, thereby inhibiting the excessive proliferation of GM [116]. Fukiya et al. [117] showed that *Bacteroides enterica* and *Escherichia coli* were involved in the production of secondary BAs in the colon, and exercise may increase the level of secondary BAs by increasing the contents of beneficial bacteria. Meanwhile, Meissner et al. [118] also revealed that compared with the mice in sedentary group, mice participating in 12-week autonomous exercise had the increased secretion of BAs and output of fecal BAs, and the fecal BAs increased with the enhancement of exercise amount and exercise intensity. On the basis of this, Clark et al. [119] showed that secondary BAs produced by GM could participate in the regulation of reactive oxygen species (ROS) and inflammatory responses in body by attenuating TNF-α-mediated immune responses and reducing the expression of inflammasomes (such as NOD-like receptor thermal protein domain associated protein 3 (NLRP3)). In addition, the dysbiosis of GM may also decrease the activation of BAs and reduce the levels of free BAs and secondary BAs, and the activation of farnesoid X receptor (FXR), takeda G protein-coupled receptor 5 (TGR5) and the interaction with mitochondria might also be weakened, and these receptors have been verified to be closely related to the regulation of the balance between osteoblasts and osteoclasts [120, 121]. It is recognized that the changes of GM and its metabolites under different intervention conditions, as well as the further in-depth regulatory effects, have become one of vital potential mechanisms of exercise-mediated OP benefits.

### The regulation of intestinal mucosal barrier, inflammatory response and oxidative stress

Different exercise amount and exercise intensity may have different effects on the intestinal mucosal barrier and mucosal immune function, and regular moderate exercise may reduce the incidence of infection in body [122]. Meanwhile, moderate exercise can reduce the intestinal inflammatory reaction without affecting the normal tissue structure and morphology of intestinal mucosal barrier [123]. Both prolonged endurance exercise and high intensity exercise could result in the impaired intestinal mucosal barrier function, and mechanisms may be related to the redistribution of blood in whole body. During the process of exercise, blood is mainly concentrated in the heart and skeletal muscles, and the symptoms (such as ischemia and hypoxia) may appear in intestine for a short period of time [124]. With the enhancement of exercise intensity and prolonged duration, the blood redistribution of body may become more apparent. Furthermore, the decrease of intestinal PH value caused by mesenteric ischemia, hypoxia and ischemia-reperfusion is the main reason to induce the intestinal oxidative stress and metabolic abnormalities. The production of excessive nitrogen and oxides caused by high intensity exercise might exacerbate the oxidative damage of intestinal biomolecules, resulting in the injury of intestinal tight junction proteins, and the structure and function of intestinal epithelial cells, thereby further resulting in “intestinal leakage” [125, 126]. Moreover, during the process of acute/vigorous exercise, core temperature in body increases continuously, and prolonged overheating (> 40 °C) can bring about the damage to intestinal epithelial cells, thereby resulting in cell shedding, intestinal villus contraction, edema or hemorrhage [127].

In addition, the long-term aerobic exercise can enhance the adaptability of body to exercise, and the intestinal mucosal barrier function can also be significantly improved under the long-term exercise intervention [128]. Kang et al. [129] indicated that long-term round-running exercise intervention could significantly increase the expression of antioxidant enzymes, anti-inflammatory factors and anti-apoptotic proteins in intestinal lymphocytes of mice, accompanied by the decline of serum inflammatory factor levels, suggesting that long-term aerobic exercise plays a positive role in maintaining intestinal villus morphology and improving intestinal permeability. Motiani et al. [130] observed that after 12 weeks of HFD induction, the basal part of intestinal villi of mice could be significantly widened, and its mechanisms may be related to the infiltration of intestinal inflammatory cells and increase of adipocytes. However, the morphology of intestinal villi and proportion of intestinal inflammatory cells in mice treated with HFD combined with long-term round-running exercise intervention were significantly improved. Other researches have also proposed that the positive impact of long-term aerobic exercise on intestinal mucosal barrier function is associated with the improvement of structure and abundance of GM, the decline of serum inflammatory level and the tolerance level of intestinal ROS [131, 132]. A large number of free radicals generated during exercise are related to the damage of bone, skeletal muscle and the decline of exercise capacity. Excessive free radicals generated by long-term and high intensity exercise might result in the body to generate the exercise-induced oxidative stress damage, which is detrimental to the health and exercise capacity of athletes [133, 134]. Hsu et al. [135] revealed that the endurance exercise time of specific pathogen free (SPF) mice was significantly higher than that of GF mice, and levels of serum glutathione peroxidase and catalase were also higher than that of GF mice, indicating that GM might eliminate the excessive free radicals produced by exercise, alleviate the exercise fatigue and improve the exercise capacity by enhancing the level of antioxidant enzymes. As there are few relevant studies, more animal models and its potential mechanisms still need to be verified in the future. Nevertheless, as a beneficial stimulus, it is recognized that the exercise intervention can enhance the long-term elasticity of intestinal mucosal barrier, reduce the release of pro-osteoclastogenic cytokines, and increase antioxidant capacity, thus inhibiting the activation of RANKL/OPG/RANK pathway, regulating the balance of osteoblasts and osteoclasts, and further preventing or treating OP.

### The involvement of immune regulation

On one hand, GM is involved in immune maturation and anti-infection protection of the body, and in this process, the microorganisms are involved in immune system to recognize and distinguish between the beneficial and harmful bacteria [136]. On the other hand, GM can directly or indirectly influence the function of dendritic cells and macrophages, modulate the activity of T cells, and induce the maturation of B cells via epithelial cells [137, 138]. Moreover, GM can also promote the immune progress of intestinal mucosa through dialogue with the immune system, which might be a critical mechanism for body to prevent the invasion of pathogens [139]. The innate immune system recognizes and differentiates the pathogens and harmless substances through the toll-like receptors (TLRs) and nucleotide-bonded oligomerization domain receptor mechanisms, while microorganisms regulate the expression of TLRs through microbe-associated molecular patterns (MAMPs) pathway, trigger the cascade effect, and further induce the immune response [140, 141].

**Fig. 2:**
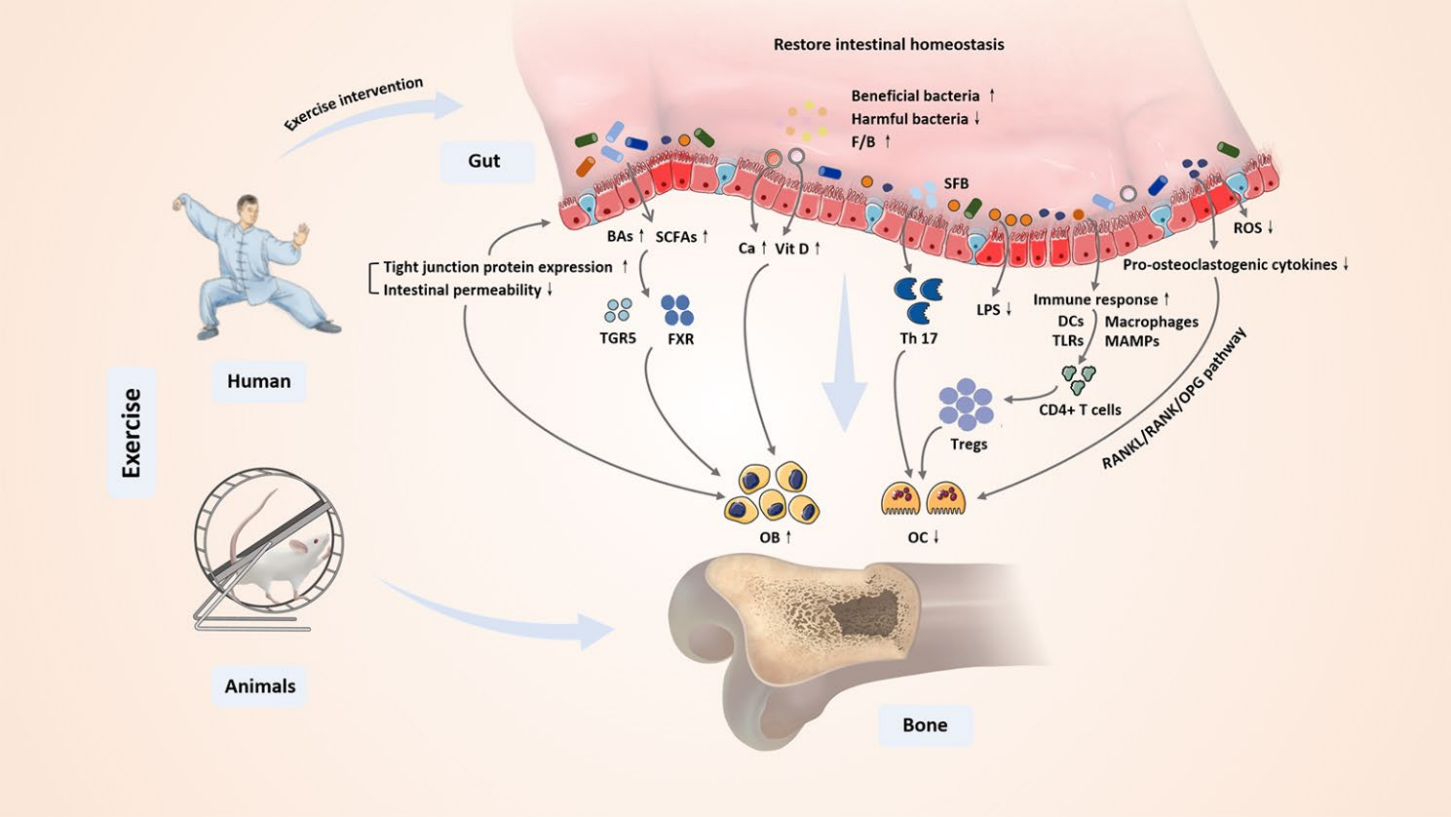
The potential mechanisms of exercise intervention on bone metabolism from the perspective of GM and its metabolites. BAs, bile acids; SCFAs, short chain fatty acids; TGR5, takeda G protein-coupled receptor 5; FXR, farnesoid X receptor; Vit D, vitamin D; F/B, *Firmicutes*/*Bacteroides*; Th 17, T helper cell 17; Tregs, regulatory cells; SFB, segmental filamentous bacteria; LPS, lipopolysaccharide; DCs, dendritic cells; TLRs, toll-like receptors; ROS, reactive oxygen species; MAMPs, microbe-associated molecular patterns; RANKL, receptor activator of nuclear factor-κ B ligand; RANK, receptor activator of nuclear factor-κ B; OPG, osteoprotegerin; OB, osteoblast; OC, osteoclast

In addition, the supplementation of probiotics/prebiotics also has a crucial impact and repercussion on exercise-mediated immune regulation. Athletes might also benefit from the regular probiotic supplementations, while with certain strain specificity [142, 143]. Probiotics commonly used to improve the immune function of athletes include *Lactobacillus* and *Bifidobacterium* [144, 145]. Previous studies have indicated that after the regular supplementation of probiotics, the severity of gastrointestinal diseases and incidence rate of respiratory diseases in high intensity male athletes are significantly reduced, and the immune disturbance induced by exercise is correspondingly weakened [146–148]. Bermon et al. [149] revealed that the course and severity of upper respiratory tract infection were decreased in the 20 high-level long-distance runners after the oral administration of *Lactobacillus yeast*, while the levels of IgA, IL-4 and IL-12 in saliva were not significantly changed. Furthermore, as a kind of indigestible polysaccharide, the prebiotics can stimulate the activation and growth of one or more beneficial bacteria in intestine. Similarly, Drakoularakou et al. [150] revealed in a randomized controlled study involving 159 healthy volunteers engaged in an international tourism that the incidence rate and the duration of diarrhea were reduced after the supplementation of *Galactooligosaccharides* (GOS). Regular supplementation of *Fructooligosaccharides* (FOS) is also related to increased serum intestinal peptide concentrations and decreased circulating C-reactive protein (CRP) levels [151]. As a result, although there are still few studies on the involvement of exercise in osteoimmunology regulation via GM and its metabolites [152], more and more relevant researches in recent years have indicated that GM has a potential immunomodulatory function and can build a bridge between exercise and osteoimmunology, indicating a novel research direction of exercise-bone-immunology. Collectively, the potential mechanism of exercise intervention on bone metabolism from the perspective of GM and its metabolites are summarized in Fig. 2.

## Conclusions and perspectives

In general, there is increasing evidence that exercise is an independent factor of external environmental stressors that affects the composition and diversity of GM, and improves the body metabolism and immune system. The imbalance of GM has a direct impact on the normal physiological function and the health status of host. Exercise can enrich the abundance and diversity of GM, improve the proportion of *Firmicutes* and *Bacteroidetes*, induce the proliferation of beneficial bacteria and metabolites, and improve the function of intestinal mucosal barrier, so as to further modulate the bone metabolism. Hence, the appropriate exercise has indelible effects on prevention and treatment of OP. However, there are still various issues need to be negotiated in the researches on relationship between exercise, bone metabolism and GM and its metabolites, specifically including: (1) The mechanisms of exercise-related GM and its metabolites directly regulating bone metabolism needs to be further improved; (2) Whether the exercise-related GM and its metabolites have the linkage effects with other important organs in body to indirectly modulate bone metabolism; (3) Paying attention to and screening out the specific GM suitable for different races around the world and the specific flora for improving the motor function may provide the novel insights for the healthy development of motor system and the improvement of motor function level. Nevertheless, with the increasing maturity of microbiome technologies and the wide application of sterile or GF animals, it is believed that further in-depth exploration might provide new perspectives for the researches on link between exercise, GM and its metabolites, and bone metabolism, and also promote the interdisciplinary and integrated researches of exercise physiology, microbiology and metabolomics.

## Electronic supplementary material

Below is the link to the electronic supplementary material.

Supplementary Material 1

Supplementary Material 2

## Data Availability

The data used during this current study are available from the corresponding author on reasonable request.

## Declarations

**Contributor information**.

Yuan-Wei Zhang, E-mail: zyw812726327@163.com.

Mu-Min Cao, E-mail: 790214357@qq.com.

Ying-Juan Li, E-mail: liyingjuan228@126.com.

Xiang-Xu Chen, E-mail: chenxiangxu0323@126.com.

Qian Yu, E-mail: hyyq1972@163.com.

Yun-Feng Rui, E-mail: ruiyunfeng@126.com.

## Abbreviations

OP: osteoporosis
GM: gut microbiota
BMD: bone mineral density
OVX: ovariectomy
GF: germ-free
TNF-α: tumor necrosis factor-α
SFB: segmental filamentous bacteria
Th 17: T helper cell 17
IL-1β: interleukin 1β
RANKL: receptor activator of nuclear factor-κ B ligand
IGF-1: insulin-like growth factor-1
P1NP: procollagen type I N-terminal propeptide
WHO: World Health Organization
SCFAs: short chain fatty acids
HPA: hypothalamic-pituitary-adrenal
ZO-1: zonula occludens-1
HFD: high-fat diet
LPS: lipopolysaccharide
LFD: low-fat die.
FMT: fecal microbiota transplantation
HSP70: heat shock protein 70
BAs: bile acids
ROS: reactive oxygen species
NLRP3: NOD-like receptor thermal protein domain associated protein 3
SPF: specific pathogen free
FXR: farnesoid X receptor
TGR5: takeda G protein-coupled receptor 5
TLRs: toll-like receptors
MAMPs: microbe-associated molecular patterns
CRP: C-reactive protein
GOS: Galactooligosaccharides
FOS: Fructooligosaccharides
SESN2: sestrin-2
CRTC2: CREB regulated transcription coactivator 2
NAFLD: non-alcoholic fatty liver disease
16S rRNA: 16 S ribosomal RNA
