# Supplementary material for: A narrative review of the moderating effects and repercussion of exercise intervention on osteoporosis: ingenious involvement of gut microbiota and its metabolites
Source: J Transl Med. 2022 Oct 27;20:490. doi: 10.1186/s12967-022-03700-4 (PMC9615371; doi:10.1186/s12967-022-03700-4)
Supplement: Supplementary file 1 — Supplementary Material 1 [file 12967_2022_3700_MOESM1_ESM.docx]

**Journal of Translational Medicine**

**Date: Oct 2, 2022**

**Ref.: Manuscript ID JTRM-D-22-03674 (Review)**

A narrative review of the moderating effects and repercussion of exercise intervention on osteoporosis: ingenious involvement of gut microbiota and its metabolites

Dear Prof. Francesco M. Marincola,

First of all, we would like to thank you and all the reviewers for thoughtful comments and your time for reviewing our manuscript.

We have currently improved the manuscript by addressing all of reviewers’ and editors’ comments and indicated how their suggestions have been incorporated in the revised manuscript when it is appropriate. A point-by-point response to comments is attached below, and the changes to this revision are uploaded in supplementary files.

We believe the revised version of the manuscript represents a significant improvement, and your favorable consideration would be greatly appreciated.

Sincerely yours,

Dr Yun-Feng Rui, Department of Orthopaedics, Zhongda Hospital, School of Medicine, Southeast University, Nanjing, Jiangsu, China. (On behalf of all co-authors)

E-mail: ruiyunfeng@126.com

**Reviewer reports:**

**Reviewer #1: In this review, the authors aimed to discuss the moderating effects and potential mechanisms of an exercise intervention on GM and bone metabolism, as well as the interaction between them.**

Reply: We thank reviewer for carefully reading our manuscript and raising recognition. After integrating the opinions of other reviewers, we have addressed all the reviewers’ questions and suggestions, and further fully supplemented previous version in revised manuscript. Thanks for the reviewers’ continued help and efforts to improve the quality of our manuscript. We believe this revised version represents a significant improvement, and your favorable consideration would also be greatly appreciated.

**Comments:**

**1. I recommend not using precise statements such as "From the perspective of exercise intervention on GM, this review may provide certain reference for the related researches to prevent and treat OP in the future" throughout the study.**

Reply: We thank the reviewer for carefully reading our manuscript and raising effective suggestions. This is indeed a point of concern. As suggested by the reviewer, we have removed these statements in the revised manuscript. Thanks for reviewer’s continued help and efforts to improve the quality of our manuscript.

**2. There are too many grammatical and spelling errors throughout the work. The authors should revise their manuscript carefully regarding typos and grammar. In addition, some sentences are very long, it may be more appropriate to express these sentences in shorter or in two sentences. Because it is important that the "review article" is written in a clean language. The study needs to be carefully reviewed on these issues.**

Reply: We thank the reviewer for your kind comment and positive advice. This is really a constructive advice, and is of great significance. Firstly, we have re-examined overall manuscript and corrected the potential spelling and grammar errors throughout the text. Secondly, we have also revised the long sentences in shorter or in two sentences in the revised manuscript. Thanks for the reviewer’s continued help and efforts to improve the quality of our manuscript.

**The manuscript needs to be revised regarding the mentioned issues before publication.**

Reply: Thanks again for the reviewer’s continued help and efforts to improve the quality of our manuscript. We believe this revised version represents a significant improvement, and your favorable consideration would also be greatly appreciated.

**Reviewer #2: Congratulations on your review. It is an interesting review where they describe the impact of exercise on the microbiota and on the synthesis of osteoclasts. It is more focused on the benefit of exercise and the microbiota and as a side effect the prevention of osteoporosis.**

Reply: We thank reviewer for carefully reading our manuscript and raising recognition. After integrating the opinions of other reviewers, we have addressed all the reviewers’ questions and suggestions, and further fully supplemented previous version in revised manuscript. Thanks for the reviewers’ continued help and efforts to improve the quality of our manuscript. We believe this revised version represents a significant improvement, and your favorable consideration would also be greatly appreciated.

**Reviewer #3: Authors are recommended to point out he following aspects:**

**Cardio vascular disease and osteoporosis together account for most of the morbidity and mortality in our aging population despite significant improvements in treatment. Recently, converging lines of evidence suggest that these 2 diseases share an etiologic factor -that hyperlipidemia contributes not only to atherosclerotic plaque formation, but also to osteoporosis, following a similar biologic mechanism involving lipid oxidation….as evident in… Role of lipids in osteoporosis. Arterioscler Thromb Vasc Biol. 2000 Nov;20(11):2346-8. doi: 10.1161/01.atv.20.11.2346. PMID: 11073836.**

Reply: We thank the reviewer for carefully reading our manuscript and raising effective suggestions. Indeed, cardiovascular diseases and osteoporosis are closely related in the etiology and pathogenesis. As suggested by the reviewer, we have added these related contents in the revised manuscript. Accordingly, the reference listed by the reviewer has also been added. Thanks for the reviewers’ continued help and efforts to improve the quality of our manuscript.

**But, dyslipidemia is a major cause of NAFLD. NAFLD and osteoporosis are strictly linked, as more recently it has been evidenced, i.e., Non-alcoholic fatty liver disease (NAFLD) is associated with an increased incidence of osteoporosis and bone fractures. Z Gastroenterol. 2022 Aug;60(8):1221-1227. English. doi: 10.1055/a-1482-9236. Epub 2021 Oct 28. PMID: 34710938.**

Reply: We thank the reviewer for carefully reading our manuscript and raising effective advice. As suggested by the reviewer, we have added the related contents in the revised manuscript. Accordingly, the reference listed by reviewer has also been added. Thanks for the reviewers’ continued help and efforts to improve the quality of our manuscript.

**Although the mechanisms remain poorly understood, it is reasonable to conclude that low levels of physical activity lead to a decrease in bone mineral density, the development of insulin resistance, and as a consequence to the presence of NAFLD..as evident in …. Novel insights into the relationship between nonalcoholic fatty liver disease and osteoporosis. Clin Interv Aging. 2018 Oct 4;13:1879-1891. doi: 10.2147/CIA.S170533. PMID: 30323574; PMCID: PMC6174895.**

Reply: We thank the reviewer for carefully reading our manuscript and raising effective advice. As suggested by the reviewer, we have added the related contents in the revised manuscript. Accordingly, the reference listed by reviewer has also been added. Thanks for the reviewers’ continued help and efforts to improve the quality of our manuscript.

**Authors correctly state that…. Goto et al. indicated that transplanting segmental filamentous bacteria (SFB) into GF mice could increase the number of T helper cell 17 (Th17) cells, resulting in the enhancement of the levels of TNF-α, interleukin 1β (IL-1β), and IL-17, and inducing the expression of receptor activator of nuclear factor-κ B ligand (RANKL), thereby playing a role in promoting the formation of osteoclasts… emphasizing the main role of the interleukin -17.**

**But interleukin 17 plays a key role in atherosclerosis following obesity related-NAFLD, as evident in...Is serum Interleukin-17 associated with early atherosclerosis in obese patients? J Transl Med. 2014 Aug 6;12:214. doi: 10.1186/s12967-014-0214-1. PMID: 25092442; PMCID: PMC4256548.**

Reply: We thank the reviewer for carefully reading our manuscript and raising effective advice. As suggested by the reviewer, we have added the related contents in the revised manuscript. Accordingly, the reference listed by reviewer has also been added. Thanks for the reviewers’ continued help and efforts to improve the quality of our manuscript. We believe this revised manuscript represents a significant improvement, and your favorable consideration would also be greatly appreciated.

**Reviewer #4: A well written and useful review article.**

Reply: We thank reviewer for carefully reading our manuscript and raising recognition. After integrating the opinions of other reviewers, we have addressed all the reviewers’ questions and suggestions, and further fully supplemented previous version in revised manuscript. Thanks for the reviewers’ continued help and efforts to improve the quality of our manuscript. We believe this revised version represents a significant improvement, and your favorable consideration would also be greatly appreciated.

**The only suggestion I would give on your title to include A Narrative Review, to clearly indicate what type of review this paper is.**

**Suggestion**

**The moderating effects and repercussion of exercise intervention on osteoporosis: ingenious involvement of gut microbiota and its metabolites: A Narrative Review**

Reply: We thank the reviewer for your kind comment and positive advice. This is really a constructive advice, and is of great significance. As mentioned by the reviewer, we have added the type of review (narrative review) in the revised version. Current title is “A narrative review of the moderating effects and repercussion of exercise intervention on osteoporosis: ingenious involvement of gut microbiota and its metabolites”. Thanks for the reviewer’s continued help and efforts to improve the quality of our manuscript.
