# Supplementary material for: A narrative review of the moderating effects and repercussion of exercise intervention on osteoporosis: ingenious involvement of gut microbiota and its metabolites
Source: J Transl Med. 2022 Oct 27;20:490. doi: 10.1186/s12967-022-03700-4 (PMC9615371; doi:10.1186/s12967-022-03700-4)
Supplement: Supplementary file 2 — Supplementary Material 2 [file 12967_2022_3700_MOESM2_ESM.docx]

Dear Prof. Francesco Marincola,

We would like to submit the manuscript entitled *“A narrative review of the moderating effects and repercussion of exercise intervention on osteoporosis: ingenious involvement of gut microbiota and its metabolites”* which we wish to be considered for the publication in the ***Journal of Translational Medicine***.

Osteoporosis (OP) is a systemic bone disease characterized by the decreased bone mass and destruction of bone microstructure, which tends to result in the enhanced bone fragility and related fractures, as well as high disability rate and mortality. Exercise is one of the most common, reliable and cost-effective interventions for the prevention and treatment of OP currently, and numerous studies have revealed the close association between gut microbiota (GM) and bone metabolism recently. Moreover, exercise can alter the structure, composition and abundance of GM, and further influence the body health via GM and its metabolites, and the changes of GM also depend on the choice of exercise mode. Herein, combined with relevant studies and based on the inseparable relationship between exercise intervention-GM-OP, this review is aimed to discuss the moderating effects and potential mechanisms of exercise intervention on GM and bone metabolism, as well as the interaction between them. From the perspective of exercise intervention on GM, this review may provide certain reference for the related researches to prevent and treat OP in the future.

Neither the entire paper nor any part of its content has been published or accepted elsewhere. It is not being submitted to any other journal.

Thank you very much for your considering our manuscript for potential publication. I’m looking forward to hearing from you soon.

Sincerely yours,

Corresponding author:

Dr Yun-Feng Rui, Department of Orthopedics, Zhongda Hospital, School of Medicine, Southeast University, Nanjing, Jiangsu, China.

E-mail: ruiyunfeng@126.com
